# Immune-Correlates Analysis of an HIV-1 Vaccine Efficacy Trial Reveals an Association of Nonspecific Interferon-γ Secretion with Increased HIV-1 Infection Risk: A Cohort-Based Modeling Study

**DOI:** 10.1371/journal.pone.0108631

**Published:** 2014-11-04

**Authors:** Yunda Huang, Ann Duerr, Nicole Frahm, Lily Zhang, Zoe Moodie, Steve De Rosa, M. Juliana McElrath, Peter B. Gilbert

**Affiliations:** 1 Vaccine and Infectious Disease Division, Fred Hutchinson Cancer Research Center, Seattle, Washington, United States of America; 2 Public Health Science Division, Fred Hutchinson Cancer Research Center, Seattle, Washington, United States of America; 3 Department of Global Health, University of Washington, Seattle, Washington, United States of America; 4 Department of Epidemiology, University of Washington, Seattle, Washington, United States of America; 5 Department of Laboratory Medicine, University of Washington, Seattle, Washington, United States of America; 6 Department of Medicine, University of Washington, Seattle, Washington, United States of America; 7 Department of Biostatistics, University of Washington, Seattle, Washington, United States of America; French National Centre for Scientific Research, France

## Abstract

**Background:**

Elevated risk of HIV-1 infection among recipients of an adenovirus serotype 5 (Ad5)-vectored HIV-1 vaccine was previously reported in the Step HIV-1 vaccine efficacy trial. We assessed pre-infection cellular immune responses measured at 4 weeks after the second vaccination to determine their roles in HIV-1 infection susceptibility among Step study male participants.

**Methods:**

We examined *ex vivo* interferon-γ (IFN-γ) secretion from peripheral blood mononuclear cells (PBMC) using an ELISpot assay in 112 HIV-infected and 962 uninfected participants. In addition, we performed flow cytometric assays to examine T-cell activation, and *ex vivo* IFN-γ and interleukin-2 secretion from CD4^+^ and CD8^+^ T cells. We accounted for the sub-sampling design in Cox proportional hazards models to estimate hazard ratios (HRs) of HIV-1 infection per 1-log_e_ increase of the immune responses.

**Findings:**

We found that HIV-specific immune responses were not associated with risk of HIV-1 infection. However, each 1-log_e_ increase of mock responses measured by the ELISpot assay (i.e., IFN-γ secretion in the absence of antigen-specific stimulation) was associated with a 62% increase of HIV-1 infection risk among vaccine recipients (HR = 1.62, 95% CI: (1.28, 2.04), p<0.001). This association remains after accounting for CD4^+^ or CD8^+^ T-cell activation. We observed a moderate correlation between ELISpot mock responses and CD4^+^ T-cells secreting IFN-γ (ρ = 0.33, p = 0.007). In addition, the effect of the Step vaccine on infection risk appeared to vary with ELISpot mock response levels, especially among participants who had pre-existing anti-Ad5 antibodies (interaction p = 0.04).

**Conclusions:**

The proportion of cells, likely CD4^+^ T-cells, producing IFN-γ without stimulation by exogenous antigen appears to carry information beyond T-cell activation and baseline characteristics that predict risk of HIV-1 infection. These results motivate additional investigation to understand the potential link between IFN-γ secretion and underlying causes of elevated HIV-1 infection risk among vaccine recipients in the Step study.

## Introduction

The Step study was a phase 2b randomized double-blind clinical trial of a preventive human immunodeficiency virus type 1 (HIV-1) vaccine in 3000 participants. It aimed to evaluate whether the adenovirus serotype 5 (Ad5)-vectored MRKAd5 HIV-1 gag/pol/nef vaccine administered at weeks 0, 4 and 26 could reduce either HIV-1 infection rates or plasma viremia after infection. This study showed no evidence for vaccine efficacy. Surprisingly, risk for HIV-1 infection was elevated among male vaccine recipients who had pre-existing Ad5 neutralizing antibodies and/or were uncircumcised [1], [2]. Several hypotheses have been raised on the mechanisms for possible vaccine-associated increased risk. For example, HIV-specific CD4^+^ T cells induced by the Step vaccine may have preferentially served as susceptible target cells for HIV-1 infection, or pre-existing Ad5-specific immunity could have played a role in HIV-specific immune responses and risk of HIV-1 infection. An initial descriptive case-cohort analysis of the vaccine-induced immunity in Step was previously reported, but found vaccine-induced HIV-specific immune responses did not correlate with risk of HIV-infection based on an earlier incomplete dataset [3]. In a related non-human primate study, a greater risk of infection was also observed in animals pre-exposed to Ad5 and immunized with an Ad5 simian immunodeficiency virus (SIV) vaccine, compared to those not pre-exposed to Ad5 [4]. Although a dampening effect of Ad-specific CD4^+^ T-cell responses on ensuing vaccine insert-specific responses was observed in a clinical trial by Frahm et al. [5], no quantitative analysis of the association between pre-existing Ad5-specific cellular immune responses and risk of HIV-1 infection was performed in the Step study due to the limitation of relevant data.

Clinical and immunological data are now available on more than twice as many HIV-1-infected and uninfected Step participants than previously described [3]. We have measured post-vaccination cellular immunity from almost all male vaccine recipients, in addition to a subset of male placebo recipients [6]. We focused the examination of *ex vivo* interferon-γ (IFN-γ) secretion in an ELISpot assay using peripheral blood mononuclear cells (PBMC) obtained at the pre-infection primary immunogenicity time-point, 4 weeks after the second vaccination. We also used flow cytometric assays to examine T-cell activation, as well as *ex vivo* IFN-γ and interleukin-2 (IL-2) secretion from different cell populations in a subset of samples.

As the first proof-of-concept efficacy trial of a cell-mediated immunity HIV-1 vaccine, the Step Study provides unique data to investigate the roles of cellular responses in predicting trial outcomes. In this study, we evaluated quantitative immune correlates based on the comprehensive up-to-date data from the Step study and its follow up study, HVTN 504 [2]. Such immune correlates analyses have not been reported in any of the three cell-mediated immunity HIV-1 vaccine efficacy trials that have been conducted so far [1], [7], [8]. We addressed the hypothesis that cellular immunity, either specific to HIV-1 proteins or non-specific, was predictive of HIV-1 infection risk in vaccine or placebo recipients, and was predictive of the vaccine effect (vaccine versus placebo) on HIV-1 infection. Our analysis of immune correlates identifies markers for HIV-1 susceptibility and provides immunological insights into the vaccine-associated enhancement of HIV-1 infection risk observed in subgroups of Step study vaccine recipients.

## Materials and Methods

### Step/HVTN504

The Step study (ClinicalTrials.gov number: NCT00095576) was a 3,000 person phase 2b efficacy trial of a recombinant Ad5-vectored HIV-1 vaccine containing *gag*, *pol*, and *nef* genes [1]. Study participants were randomly assigned in a 1∶1 ratio to receive three doses of the vaccine or placebo on day 1 (study enrollment), week 4, and week 26. The placebo was vaccine diluent only. The initial planned interim analysis met the pre-specified futility criteria for efficacy [1]. Subsequently, vaccinations were halted and participants unblinded. To assess the longer-term risk of HIV-1 infection, Step participants were enrolled in HVTN 504 for extended follow-up of up to four years from the time of first vaccination [2]. All volunteers provided informed written consent prior to Step study participation, and the institutional review boards at the Fred Hutchinson Research Center approved the described study.

### Laboratory assays

A validated *ex vivo* IFN-γ ELISpot assay [9] was the primary immunological assay to quantify the number of IFN-γ secreting cells in previously cryopreserved PBMC collected at week 8, 4 weeks after the second vaccination [3]. HIV non-specific mock responses served as assay negative control and were obtained as the number of spot-forming cells (SFC) per million PBMC in the absence of any antigen stimulation (media only). The number of SFC per million PBMC after stimulation with pools of 15-mer peptides from the HIV-1 antigens Gag, Pol and Nef were also determined. Average SFC from three replicates of antigen-stimulated responses or six replicates of mock responses were used in subsequent analyses.

Flow cytometric assays were performed to quantify the percent of CD4^+^ and CD8^+^ T cells with surface expression of Ki-67^hi^BcL-2^lo^ as a measure of antigen non-specific T-cell activation using week 8 PBMC. A validated *ex vivo* intracellular cytokine staining (ICS) assay was also performed to measure the percent of HIV-, CMV- and Ad5-specific CD4^+^ and CD8^+^ T cells secreting IFN-γ and/or IL-2 using week 8 PBMC. ICS mock responses were obtained using dimethyl sulfoxide (DMSO, the peptide diluent) as assay negative control [10]. ICS mock responses of IFN-γ production in CD4^+^ T cells, CD8^+^ T cells, singlets, live cells, CD3^+^CD4^−^CD8^−^ (double negative CD3^+^) cells, and CD3^−^ cells were also obtained in a subset of samples. Unless otherwise stated, all antigen-specific ELISpot and ICS responses were determined as the difference between antigen-stimulated responses and the negative control (mock response). Immune responses without subtracting off negative control values were also examined and referred to as background-unadjusted responses. High-resolution HLA class I typing (4 digits; HLA–A, HLA–B, and HLA–C) was obtained using sequence-based methods [6].

### Statistical Analysis

Because only 15 HIV-1 infections were detected among female participants [2], all reported analyses were restricted to male participants in the per-protocol (PP) cohort as previously defined [1]. We assessed immune responses at week 8 in vaccine and placebo recipients who acquired HIV-1 infection after week 12 and compared them with responses in vaccine and placebo recipients who did not acquire infection over a follow-up period of 48 months. We used Cox regression models to estimate hazard ratios (HRs) of HIV-1 infection per 1-log_e_ increase of immune responses, as well as the two-way interactions between vaccine assignment (vaccine or placebo) and immune responses, and the three-way interactions between vaccination assignment, immune responses, and either baseline Ad5 seropositivity or circumcision status in predicting the rate of HIV-1 infection among vaccine and placebo recipients. We defined the time-to-event variable as time from the week 12 visit to estimated time of infection [2]. We examined plots of the estimated time-varying hazard ratio based on scaled Schoenfeld residuals versus logarithm of follow up time and used the Grambsch and Therneau's test [11] to assess the proportional hazard assumption for each predictor in the Cox models.

Because almost all male vaccine recipients had ELISpot responses measured, no adjustment was needed to correct for sampling bias that normally occurs in two-stage sampling design of immune correlates analyses. ELISpot responses from placebo recipients and other immune responses from vaccine recipients were measured on a stratified random sample defined before the initial Step interim analysis, as well as on samples drawn afterwards for other purposes [6]. We accommodated these sub-sampling designs using two-phase analysis with post-stratification on infection status in the Cox models [12]. Post-stratification on both vaccination assignment and infection status was used for the analysis of interactions between vaccination assignment and immune responses overall and in subgroups [13]. We also conducted these analyses without post-stratification in the two-phase analysis, as well as model-based weighted analyses with empirical sampling probability (data not shown given similar results). Unless otherwise indicated, all models adjusted for potential baseline confounding factors [2], including circumcision status (yes or no), Ad5 serostatus (positive or negative, defined as having a serum neutralizing antibody titer of >18 or ≤18), region (North America + Australia or other), race (white or other), age (≤30 or >30), HSV-2 serostatus (positive or negative), and risk behavior in the past 6 months: recreational drug usage (yes or no), unprotected receptive anal sex with male partners (yes or no), unprotected insertive anal sex with HIV+ male partner (yes or no), and number of male partners ( ≤4 or >4). When a significant immune correlate was observed, we also adjusted for HLA class I type (Protective: expressing HLA–B*57, -B*58∶01 or -B*27 in at least one allele; Unfavorable: expressing HLA-B*35∶02, -B*35∶03, -B*35∶04, or -B*53∶01 in at least one allele or homozygous in at least one locus; or Neutral: remaining subjects) to examine possible confounding by host genetics among vaccine recipients.

For ELISpot responses, we applied individual-level data normalization to correct for inter-laboratory assay variability since one subset of samples were assayed by the Merck research lab, and another subset with partial overlap by the HIV Vaccine Trials Network (HVTN) central lab [14] (Figure S1 in Information S1). We also conducted analyses stratified by lab (data not shown given similar results). To better satisfy the modeling assumptions, we transformed ELISpot response data to the natural log scale during the normalization process and in all subsequent statistical analyses. To account for dependency of the normalized assay values, we used bootstrap-based estimates of standard errors and confidence intervals for inferences in all subsequent analyses that included ELISpot mock responses in the model, each with 2000 simulated datasets. All other immune response data were solely generated by the HVTN central lab, and no between-lab normalization or bootstrap-based inferences were needed in subsequent analyses.

We used univariate and multivariate linear regression models to examine baseline factors that may be predictive of the ELISpot responses. We adopted the all subset model selection procedure based on the Akaike information criterion (AIC) in building the multivariate models including possible two-way interactions between vaccination assignment and other covariates. We accommodated the post-hoc sampling design using two-phase analysis with post-stratification on both vaccination assignment and infection status in the selected models. Two-sided p-values less than 0.05 were considered statistically significant. We performed all analyses using R software (version 2.5.0).

## Results

**Figure 1:**
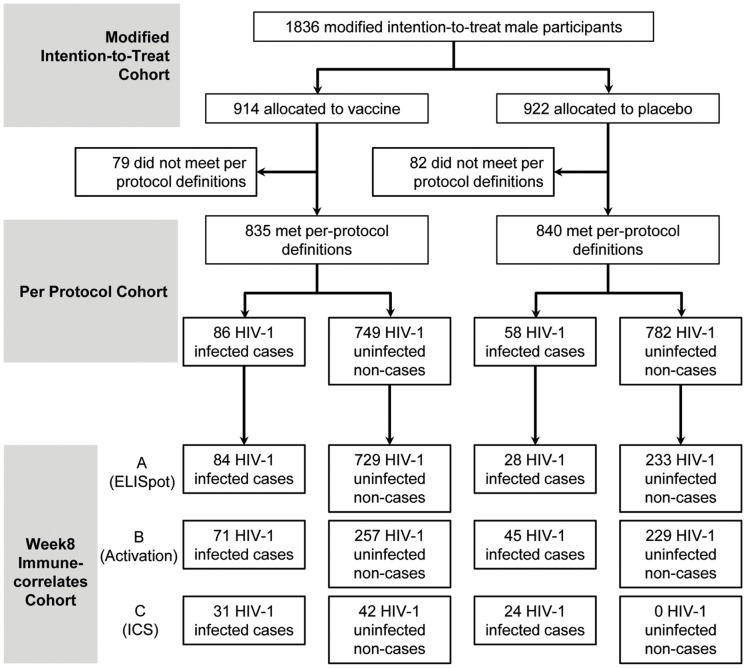
Week 8 Samples for the Immune-Correlates Analysis. Rows A, B and C indicate available data from the ELISpot, T-cell activation and ICS assays, respectively. We used the ELISpot assay to measure non-HIV-specific (mock) and HIV-specific (Gag, Pol and Nef) ex vivo IFN-γ-secreting PBMC, the T-cell activation assay to measure CD4^+^ and CD8^+^ T-cell activation marked by Ki-67^hi^BcL-2^lo^, and the ICS assay to measure CMV- and HIV-specific (Gag, Pol and Nef) *ex vivo* IFN-γ/IL-2 secretion from CD4^+^ and CD8^+^ T cells. We also measured a subset of these samples for Ad5-specific *ex vivo* IFN-γ/IL-2 secretion from CD4^+^ and CD8^+^ T cells by the ICS assay (Figure S3 in Information S1).

**Figure 2:**
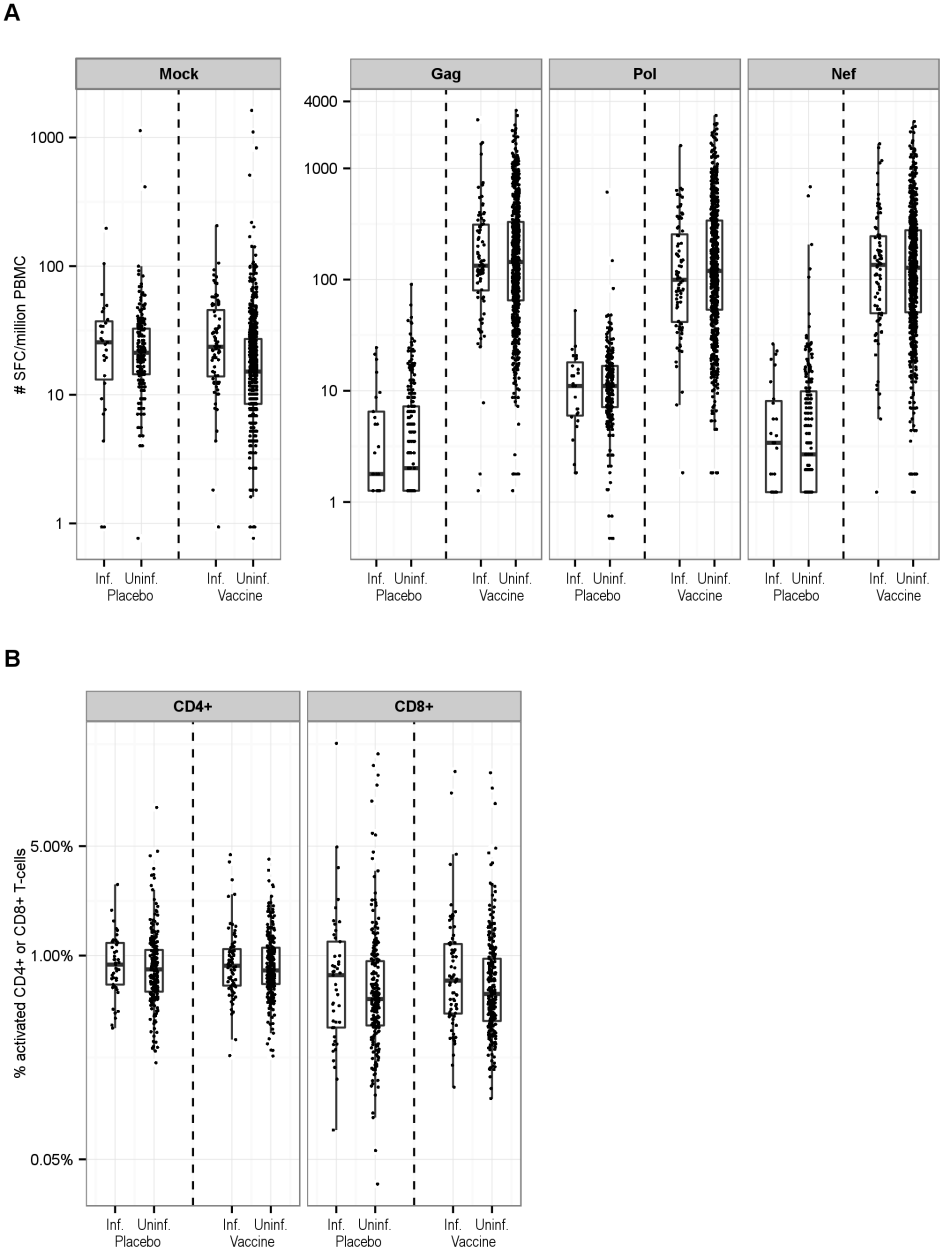
Distribution of Immune Reponses in Infected and Uninfected Vaccine and Placebo Recipients in the Immune Correlates Analysis Study. Panel A includes the IFN-γ-secreting cellular responses measured by the ELISpot assay. Panel B includes T-cell activation responses measured by the flow cytometric assay. Box-plots show the 25th percentile (lower edge of the box), 50th percentile (horizontal line in the box), and 75th percentile (upper edge of the box) for the immune responses, with participants stratified according to HIV-1 infection status and treatment assignment. The tip of the vertical bars indicate the most extreme data points, which are no more than 1.5 times the interquartile range from the box. The distribution plots of other immune responses measured by the ICS assay are shown in Figures S2 of Information S1.

We assessed cellular immune responses by ELISpot in 112 (n = 84 vaccine; n = 28 placebo) HIV-1 infected cases and 962 (n = 729 vaccine; n = 233 placebo) uninfected non-cases, T-cell activation in 116 (n = 71 vaccine; n = 45 placebo) cases and 586 (n = 257 vaccine; n = 229 placebo) non-cases, as well as T-cell responses by ICS in 51 (n = 31 vaccine; n = 24 placebo) cases and 42 vaccine non-cases (Figure 1). The vaccine was immunogenic as reported previously [9]. In addition, we observed a good dynamic range in all study subjects regardless of infection status for ELISpot mock and HIV-specific responses, as well as for T-cell activation (Figure 2) and ICS responses (Figure S2 in Information S1). These immune responses in cases tended to be comparable with or higher than those in non-cases; formal comparisons are presented later in this section to account for the sampling design and potential confounding factors. Descriptively, for ELISpot, among the vaccine recipients the median (interquartile range [IQR]) responses for Gag were 172 [87, 362] (cases: 181 [118, 331]; non-cases: 170 [83, 366]) SFC/million PBMC, for Pol 164 [85, 408] (cases: 178 [96, 375]; non-cases: 162 [84, 410]) SFC/million PBMC, for Nef 152 [73, 308] (cases: 173 [84, 283]; non-cases: 150 [72, 308]) SFC/million PBMC, and for mock 16 [9, 29] (cases: 24 [14, 46]; non-cases: 15 [8, 27]) SFC/million PBMC; among the placebo recipients, the median (IQR) mock response was 22 [14, 34] (cases: 26 [13, 37]; non-cases: 21 [14, 33]) SFC/million PBMC. For T-cell activation, among the vaccine recipients the median [IQR] was 0.81% [0.65%, 1.12%] Ki-67^hi^BcL-2^l^°CD4^+^ (cases: 0.86% [0.64%, 1.10%]; non-cases: 0.80% [0.66%, 1.12%]), and 0.59% [0.39%, 1.03%] Ki-67^hi^BcL-2^l^°CD8^+^ T cells (cases: 0.69% [0.43%, 1.19%]; non-cases: 0.57% [0.38%, 0.95%]); among the placebo recipients the median [IQR] was 0.82% [0.60%, 1.11%] Ki-67^hi^BcL-2^l^°CD4^+^ (cases: 0.88% [0.65%, 1.20%]; non-cases: 0.82% [0.59%, 1.09%]), and 0.53% [0.36%, 0.94%] Ki-67^hi^BcL-2^l^°CD8^+^ T cells (cases: 0.75% [0.35%, 1.23%]; non-cases: 0.53% [0.36%, 0.92%]). For ICS, among the vaccine recipients the median [IQR] CMV-specific responses were 0.13% [0.03%, 0.62%] IL-2^+^ and/or IFN-γ^+^ CD4^+^ (cases: 0.13% [0.04%, 0.28%]; non-cases: 0.13% [0.03%, 2.20%]), and 0.76% [0.06%, 2.56%] IL-2^+^ and/or IFN-γ^+^ CD8^+^ T cells (cases: 0.84% [0.28%, 1.87%]; non-cases: 0.60% [0.05%, 3.59%]); among the placebo recipients the median (IQR) CMV-specific responses (only available in cases) were 0.11% [0.01%, 0.22%] IL-2^+^ and/or IFN-γ^+^ CD4^+^ and 0.22% [0.01%, 1.05%] IL-2^+^ and/or IFN-γ^+^ CD8^+^ T cells (Figure S2 in Information S1). Because CD4^+^ and CD8^+^ T-cell responses detected by ICS were previously reported not to be associated with risk of HIV-1 infection [3] and there were a small number of infected and uninfected participants with available data, we refrained from further quantitative analysis of ICS responses.

**Figure 3:**
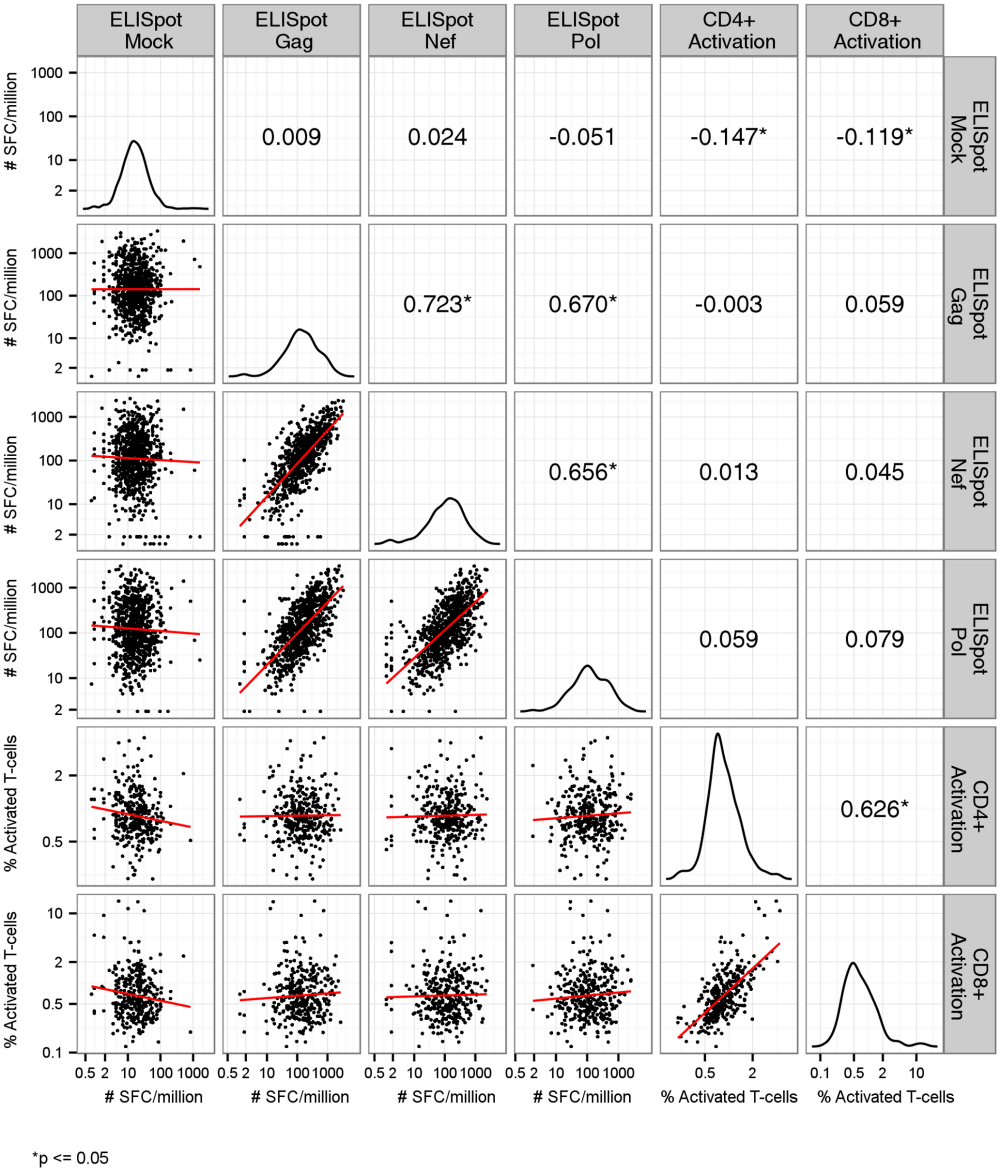
Correlations among IFN-γ-secreting Cellular Responses Measured by ELISpot, and T-cell Activation Responses among Vaccine Recipients. Values in the upper triangle indicate the Spearman's correlation coefficients for each pair of immune responses. Curves in the diagonal entries indicate the density distribution of each immune response. Plots in the lower triangle indicate the joint distribution of each pair of immune responses with a fitted simple linear regression line.

In examining the relationships between immune responses from vaccine recipients, we observed that ELISpot responses against different HIV-1 antigens were correlated with each other (Spearman correlation coefficients ρ>0.65), and T-cell activation was correlated between CD4^+^ and CD8^+^ T-cell subsets (ρ = 0.63). On the other hand, ELISpot mock responses showed no or low correlation with the HIV-specific ELISpot responses or T-cell activation (Figure 3). Among a subset of vaccine recipients in whom we measured other immune responses of interest, ELISpot mock responses also showed no correlation with Ad5-specific or CMV-specific CD4^+^ or CD8^+^ T-cells secreting IL-2 and/or IFN-γ (Figure S3 in Information S1), and no correlation with CD8^+^ T-cells, CD3^+^ CD4^−^CD8^−^ (double negative) T cells or CD3^−^ cells secreting IFN-γ (Figure S4 in Information S1). However, we observed a moderate correlation between ELISpot mock responses and ICS mock responses of CD4^+^ T-cells secreting IFN-γ (ρ = 0.33, p = 0.007), suggesting that CD4^+^ T-cells may have been the source of IFN-γ secretion in mock ELISpot (Figure S4 in Information S1).

In relating these immune responses with susceptibility to HIV-1 infection, we found that ELISpot mock responses were directly correlated with risk of HIV-1 infection among vaccine recipients. We observed an estimated 62% increase in risk of HIV-1 infection (baseline-covariate-adjusted HR = 1.62, 95% confidence interval [CI]: (1.28, 2.04), p<0.001) per 1-log_e_ increase of mock responses (Table 1). HIV-specific ELISpot responses were not significantly associated with risk of HIV-1 infection among vaccine recipients with or without adjustment for ELISpot mock responses. Similar results were obtained when examining background-unadjusted HIV-specific ELISpot responses (Table S1 in Information S1). CD4^+^ or CD8^+^ T-cell activation by itself was not significantly associated with risk of HIV infection among vaccine recipients. However, after adjustment for ELISpot mock responses, there was a trend of CD8^+^ T-cell activation being directly correlated with risk of HIV infection (HR = 1.60, 95% CI: (1.00, 2.55), p = 0.05). The effect of ELISpot mock responses on risk of HIV-1 infection remained significant after adjustment for HIV-specific ELISpot responses and T-cell activation (Table 1). In addition, the HR of ELISpot mock responses was fairly constant over time (time-varying HR p = 0.51) (Figure S5 in Information S1). We did not find HLA class I type to be a significant confounder in these analyses (Figure S6 and Table S2 in Information S1).

**Table 1:** Hazard ratios (HRs) for HIV-1 infection per 1- log_e_ increase of Immune responses among vaccine recipients.

Immune variable(s) in each Model	Immune variable	Baseline-covariate-unadjusted model*		Baseline-covariate-adjusted model**	
		HR per 1- log_e_ increase (95% CI)	p-value	HR per 1- log_e_ increase (95% CI)	p-value
**ELISpot responses**					
Mock	Mock	1.61 (1.31, 1.97)	<0.001	1.62 (1.28, 2.04)	<0.001
Gag	Gag	0.98 (0.84, 1.15)	0.80	1.01 (0.80, 1.27)	0.93
Pol	Pol	0.89 (0.76, 1.03)	0.11	0.89 (0.73, 1.08)	0.24
Nef	Nef	0.99 (0.85, 1.16)	0.94	1.04 (0.86, 1.26)	0.70
**T-cell activation responses**					
CD4^+^ T-cell activation	CD4^+^ T-cell activation	1.19 (0.67, 2.09)	0.55	1.02 (0.52, 1.99)	0.96
CD8^+^ T-cell activation	CD8^+^ T-cell activation	1.32 (0.96, 1.79)	0.09	1.32 (0.91, 1.92)	0.13
**ELISpot mock responses and HIV-specific responses**					
Mock & Gag	Mock	1.61 (1.31,1.97)	<0.001	1.62 (1.29,2.04)	<0.001
	Gag	0.98 (0.85,1.15)	0.84	0.99 (0.8,1.23)	0.94
Mock & Pol	Mock	1.61 (1.30,1.98)	<0.001	1.64 (1.31,2.04)	<0.001
	Pol	0.89 (0.77,1.03)	0.13	0.87 (0.72,1.06)	0.16
Mock & Nef	Mock	1.61 (1.30,2.00)	<0.001	1.62 (1.28,2.05)	<0.001
	Nef	1.01 (0.88,1.17)	0.87	1.04 (0.86,1.25)	0.68
**ELISpot mock responses and T-cell activation responses**					
Mock & CD4^+^ T-cell activation	Mock	1.65 (1.27,2.14)	<0.001	1.64 (1.18,2.30)	0.003
	CD4^+^ T-cell activation	1.36 (0.71,2.57)	0.35	1.24 (0.55,2.82)	0.60
Mock & CD8^+^ T-cell activation	Mock	1.67 (1.28,2.19)	<0.001	1.71 (1.20,2.44)	0.003
	CD8^+^ T-cell activation	1.46 (1.01,2.11)	0.04	1.60 (1.00,2.55)	0.05

*All unadjusted immune variable models included only the indicated immune variable(s) as predictor(s) of HIV-1 infection without adjustment of other baseline covariates.

**In addition to the indicated immune variable (s), all adjusted immune variable models adjusted for baseline covariates including circumcision status, Ad5 seropositivity, region, race, age, HSV-2 serostatus, recreational drug usage, unprotected receptive anal sex with HIV+ male partner, unprotected insertive anal sex with HIV+ male partner, and number of male partners.

Based on combined data from vaccine and placebo recipients, we observed that the effect of ELISpot mock responses on HIV-1 infection risk did not significantly differ between treatment groups overall (interaction p = 0.18, Table 2). We then examined this relationship within four subgroups defined by baseline circumcision status or Ad5 serostatus because each factor was identified as a potential vaccine effect modifier in previous analyses [1], [2]. Among uncircumcised men, there was a significant two-way interaction between vaccination and mock responses in predicting risk of HIV-1 infection (p = 0.04), suggesting the vaccine effect varied with the level of mock responses. Specifically, among uncircumcised vaccine recipients, an estimated 74% increase in risk of HIV-1 infection (HR: 1.74, 95% CI: [1.27, 2.41]) was associated with each 1-log_e_ increase of mock responses; among uncircumcised placebo recipients, however, an estimated 47% decrease in risk of HIV-1 infection (HR: 0.53, 95% CI: [0.18, 1.58]) was associated with each 1-log_e_ increase of mock responses. Breaking down the same interaction estimate in a different way by mock response quartiles, we estimated an HR (vaccine: placebo) of 0.80 (95% CI: [0.25, 2.60]) for a given mock response of 10 SFC/million PBMC, and an HR of 2.76 (95% CI: [0.55, 13.77]) for a given mock response of 27 SFC/million PBMC. The interaction between vaccination assignment and mock responses did not reach statistical significance among the other three subgroups: circumcised men, Ad5 seropositive men or Ad5 seronegative men, although there was a trend within Ad5 seropositive men (Table 2).

**Table 2:** Hazard ratios (HRs) for HIV-1 infection by vaccination assignment (3^rd^ column) and by mock response quartiles (6^th^ column).

Subgroup	Vaccination assignment	HR per 1-log increase of mock (95% CI)	Mock × vaccination assignment interaction p-value	Mock response quartile (#SFC per million PBMC)	HR vaccine: placebo (95% CI)
All men	Vaccine	1.60 (1.31, 1.97)	0.18	25% (10)	1.07 (0.60, 1.91)
				50% (18)	1.39 (1.02, 1.90)
	Placebo	1.01 (0.53, 1.93)		75% (30)	1.76 (1.20, 2.58)
Ad5 Seronegative Men	Vaccine	1.50 (1.07, 2.11)	0.78	25% (9)	1.77 (0.41, 7.59)
				50% (16)	1.56 (0.79, 3.07)
	Placebo	1.82 (0.49, 6.76)		75% (28)	1.41 (0.83, 2.38)
Ad5 seropositive Men	Vaccine	1.68 (1.27, 2.22)	0.12	25% (11)	0.92 (0.45, 1.85)
				50% (19)	1.37 (0.87, 2.15)
	Placebo	0.79 (0.32, 1.97)		75% (31)	1.99 (1.06, 3.75)
Circumcised Men	Vaccine	1.47 (1.09, 1.97)	0.96	25% (10)	1.46 (0.68, 3.14)
				50% (19)	1.47 (0.95, 2.28)
	Placebo	1.44 (0.74, 2.81)		75% (31)	1.49 (0.97, 2.29)
Uncircumcised Men	Vaccine	1.74 (1.27, 2.41)	0.04	25% (10)	0.80 (0.25, 2.60)
				50% (17)	1.49 (0.43, 5.21)
	Placebo	0.53 (0.18, 1.58)		75% (27)	2.76 (0.55, 3.77)

Results were obtained from Cox regression models with interaction terms between ELISpot mock responses and vaccination assignment overall and in subgroups.

In order to further understand the above results, we examined baseline predictors of ELISpot mock responses in multivariate analysis. Interestingly, when combining data from both treatment groups regardless of infection status, vaccine recipients had an average 0.23 log_e_ (95% CI: [0.12, 0.35], p<0.001) lower mock responses than placebo recipients, circumcised men had an average 0.14 log_e_ (95% CI: [0.20, 0.25], p = 0.02) higher mock responses than uncircumcised men, and Ad5 seropositive men had an average 0.23 log_e_ (95% CI: [0.11, 0.35], p<0.001) higher mock responses than Ad5 seronegative men (Table 3). No interaction between vaccination and either circumcision status or Ad5 serostatus was observed in predicting Week 8 mock responses (p>0.20). Among placebo recipients only, we found that circumcision status and Ad5 seropositivity were also significantly associated with mock responses. Among vaccine recipients, white men had an average 0.14 log_e_ (95% CI: [0.00, 0.28], p = 0.05) higher mock responses than non-white men, and Ad5 seropositive men had 0.20 log_e_ (95% CI: [0.06, 0.34], p = 0.01) higher mock responses than Ad5 seronegative men (Table 3). Mock responses were not different between HLA Class I types (Table S3 in Information S1).

**Table 3:** Baseline predictors of ELISpot mock responses among vaccine and placebo recipients.*

Placebo and Vaccine recipients combined		
Covariate	Coefficient (95% CI)	p-value
Circumcision (yes vs. no)	0.14 (0.20,0.25)	0.02
Ad5 serostatus (positive vs. negative)	0.23 (0.11,0.35)	<0.001
Vaccination assignment (vaccine vs. placebo)	−0.23 (−0.35,−0.12)	<0.001
**Placebo recipients only**		
Circumcision (yes vs. no)	0.26 (0.07,0.45)	0.008
Race (white vs. other)	−0.18 (−0.39,0.03)	0.10
Ad5 serostatus (positive vs. negative)	0.25 (0.05,0.45)	0.01
Recreational drugs (yes vs. no)	0.17 (−0.05.0.40)	0.13
Number of male partners (>4 vs. ≤4)	0.28 (−0.03,0.59)	0.08
**Vaccine recipients only**		
Race (white vs. other)	0.14 (0.00, 0.28)	0.05
Ad5 serostatus (positive vs. negative)	0.20 (0.06, 0.34)	0.01

*Estimates are shown for predictors identified in the best-fitting multivariate linear regression models based on the AIC model-selection criterion.

## Discussion

This immune correlates analysis provides a comprehensive examination of cellular immunity in predicting risk of HIV-1 infection, and the unique role of mock responses in predicting the effect of an HIV-1 vaccine aimed at inducing cellular responses on risk of HIV-1 infection. Among Step study participants, we found that vaccine-induced cellular immunity measured by the number of IFN-γ producing PBMC after stimulation with HIV-1 antigens was not predictive of the risk of HIV-1 infection. However, the proportion of IFN-γ producing cells in the absence of antigen stimulation, although lower among vaccine versus placebo recipients, was positively correlated with the risk of HIV-1 infection among vaccine recipients. Such a positive correlation was not found among placebo recipients. We also found that non-specific T-cell activation measured by high expression of Ki-67 and down-regulation of BcL-2 was not an independent predictor of the risk of HIV-1 infection by itself; however, after adjustment for ELISpot mock responses, CD8^+^ T-cell activation was directly correlated with risk of HIV-1 infection. Moreover, within the subgroups of uncircumcised men and Ad5 seropositive men, ELISpot mock responses appeared to have a different effect on the risk of HIV-1 infection in vaccine versus placebo recipients. Notably, these two Step study subgroups were previously reported to have an elevated risk of HIV-1 infection among vaccine versus placebo recipients [1], [2]. In terms of correlations among immune responses, we observed that the proportion of IFN-γ-producing PBMC in mock ELISpot was correlated with the proportion of IFN-γ-producing CD4^+^ T cells in mock ICS, but not with other responses described in this study.

The overall lower ELISpot mock responses observed among vaccine recipients versus placebo recipients and the positive correlation between ELISpot mock and IFN-γ-producing CD4^+^ T cells among vaccine recipients suggest that non-specific IFN-γ-producing cells, likely CD4^+^ T cells, were dampened by vaccination. This could be due to the possibility that non-specific IFN-γ production was minimized during the induction phase of immune responses to the vaccine (inserts and/or vector). Because IFN-γ plays a crucial role in the immediate and long-term combat against viral infection, IFN-γ secretion detected in mock ELISpot could be due to residual responses against prior viral infections, including but not limited to Ad5, in Step participants. This possibility is consistent with the observation that vaccine-associated increased risk of HIV-1 infection concentrated in two subgroups of Ad5 seropositive men and uncircumcised men with the highest proportion of non-specific IFN-γ-producing cells measured in mock ELISpot. Furthermore, given that ELISpot mock responses were not correlated with the vaccine-induced immune responses (inserts and vector), it is likely that ELISpot mock responses may have corresponded to an unmeasured marker for increased susceptibility to HIV-1 infection. Such an unmeasured marker, for example, could be HIV-specific activation of CD4^+^ T cells that was not captured by the expression of Ki-67 and BcL-2 assessed in this study. In summary, these observations were consistent with the hypothesis that CD4^+^ T cells could have played a role in vaccine-associated risk of HIV-1 infection in Step.

The effect of mock response remained but the effect of CD8^+^ T-cell activation became more apparent when these two immune variables were both evaluated as predictors of risk of HIV-1 infection. That is, mock responses negatively confounded the effect of CD8^+^ T-cell activation on risk of HIV-1 infection, possibly because mock responses explained variability in CD8^+^ T-cell activation. This suggests that mock responses should be controlled for in future analysis of the effect of T-cell activation on HIV-1 infection. This further supports the hypothesis that mock responses are an independent marker of increased susceptibility to HIV-1 infection with an underlying mechanism that was not adequately captured by any of the T-cell responses studied to date in the Step trial. This same mechanism, possibly through CD4^+^ T cells, may have led to an increased risk of infection among the Ad5 seropositive and uncircumcised subgroups of male vaccine recipients. This hypothesis is supported by associations seen in the subgroups of uncircumcised men, and to a lesser extent of Ad5 seropositive men, where higher ELISpot mock responses were associated with larger relative risk of HIV-1 infection (vaccine: placebo) even after controlling for other baseline covariates. These observations suggest that ELISpot mock responses, and a more comprehensive immune profile associated with these responses, may warrant study as candidate correlates of vaccine enhancement of HIV-1 acquisition risk.

Mock cellular responses, to our knowledge, have not been previously studied in terms of their effect on risk of HIV-1 infection; however, increased risk of infection associated with vaccine-specific cellular immunity has recently been discovered in several studies, including a non-human primate vaccine study against HSV infection [15] and the VAX004 study, where higher vaccine-specific CD8^+^ T-cell responses were significantly correlated with higher risk of HIV-1 infection among participants who received the AIDSVAX vaccine [16]. In addition, based on a recent microbicide HIV-1 prevention study, pre-existing immune activation or natural killer cell function were also found to be associated with increased risk of HIV-1 infection among women [17]–[19]. We thus hypothesize that the proportion of cells producing IFN-γ without stimulation by exogenous antigen may provide information on innate immune activation or natural killer cell function. The mock cellular responses measured by ICS were not found to predict the rate of HIV-1 infection in the case-control analysis of the RV144 trial [20] (data not shown). This could be due to the different mechanisms for providing protection against HIV-1 infection between the Step and the RV144 vaccine regimens, as well as the lower sensitivity of the ICS assay to detect bulk T-cell responses compared to the ELISpot assay and the much lower CD8^+^ T-cell responses induced by the RV144 vaccine regimen. It is also likely that the partial protective effect of the RV144 vaccine may mask the effects of mock responses on HIV-1 infection susceptibility.

One limitation of this analysis is that no pre-vaccination PBMC samples were available from the baseline visit. Therefore, it was not possible to evaluate whether and at what magnitude mock responses changed upon vaccination within the same study participants. On the other hand, we did observe an overall lower mock response among vaccine versus placebo recipients based on a large amount of ELISpot response data generated at post-vaccination time points. Another limitation of this study is that we included ELISpot data generated from two different laboratories. Although inter-lab differences in assay measurements have been corrected using a calibration method with satisfactory performance demonstrated in a validation dataset using Step study samples, residual inter-lab differences may still persist in the combined ELISpot response data, which could possibly influence our results. On the other hand, additional lab-specific analyses reached the same conclusions, although with less precision due to not combining assay results from the two labs (data not shown). These confirmative analyses used mock responses from each lab without any data calibration as predictors of HIV-1 infection risk. Lastly, due to the limitation of the ELISpot assay and the limited vaccine insert-specific and vector-specific ICS data that are available at the described time-point especially for infected vaccine recipients, we were not able to pinpoint which mononuclear cell in peripheral blood manifested the described associations and whether the vector-specific responses played a role. On the other hand, we observed some evidence for the possible role of non-specific IFN-γ-secreting CD4^+^ T cells in explaining the vaccine-associated increased risk of HIV-1 infection in Step.

Although cellular immunity was not found to be significantly predictive of the rate of HIV-1 infection in RV144, the only other vaccine efficacy trial where a quantitative immune correlate analysis was conducted [20], it is important to understand the role of cellular immunity, especially in cell-mediated vaccine trials where increased risk of HIV-1 infection was observed in subgroups of vaccine recipients. We performed a comprehensive analysis of the available immunological and host genetics data, including HIV non-specific and HIV-specific ELISpot responses to the vaccine insert, CD4^+^ and CD8^+^ ICS responses to the vaccine insert, the vaccine vector and CMV, non-specific CD4^+^ and CD8^+^ T-cell activation, and HLA data with complete ascertainment of infections in Step and its long-term follow up study. The underlying mechanism for the observed correlation between ELISpot mock responses and risk of HIV-1 infection among vaccine recipients is not entirely clear; therefore, these results motivate additional research to understand the potential link between IFN-γ secretion, especially from CD4^+^ T cells, and underlying causes of vaccine-associated enhanced infection risk in Step study participants.

## Supporting Information

Information S1 **Supporting figures and tables.** Figure S1. Week 30 paired ELISpot responses to Gag in the assay comparison study (Panel A & B) and week 8 ELISpot responses to Gag in the Main study before and after calibration (Panel C & D). Panels A & B show the distribution of Gag responses based on the paired samples. One outlier of Gag response was removed before the calibration. Panel C shows the distribution of the 27 bonus samples before and after calibration. Panel D shows the distribution of the Week 8 samples before and after calibration. Figure S2: Distribution of ICS Reponses in Infected (Inf.) and Uninfected (Uninf.) Vaccine and Placebo Recipients. Panel A includes the mock ICS responses. Panel B includes the ICS responses against CMV stimulation. Panel C includes the ICS responses against HIV-1 antigens. Panel D includes the ICS responses against Ad5 antigens. Box plots show the 25th percentile (lower edge of the box), 50th percentile (horizontal line in the box), and 75th percentile (upper edge of the box) for the immune responses, with participants stratified according to HIV-1 infection status and treatment assignment. The tip of the vertical bars indicate the most extreme data points, which are no more than 1.5 times the interquartile range from the box. Figure S3: Correlations between Mock ELISpot Responses and Ad5-specific or CMV-specific ICS Responses among Vaccine Recipients. A fitted simple linear regression line is added with values in the upper corner indicating the Spearman's correlations coefficients (ρ) and p-values (p) from the exact test of ρ being zero. Figure S4: Correlations between the Number of IFN-γ-secreting Cells Measured by Mock ELISpot and the Proportions of IFN-γ-secreting Cells in Different Cell Subsets Measured by Mock ICS among Vaccine Recipients. A fitted simple linear regression line is added with values in the upper corner indicating the Spearman's correlations coefficients (ρ) and p-values (p) from the exact test of ρ being zero. Figure S5: Estimated instantaneous hazard ratios over time per 1-log_e_ increase of immune responses from each baseline-covariate-adjusted model of a single immune variable as presented in Table 1. Figure S6: Distribution of Week 8 mock ELISpot responses by HLA class I categories among vaccine recipients. Red dots indicate infected cases and blue dots indicate uninfected non-cases. Mock responses do not seem to differ among different HLA class I categories. As shown in Table S3 in Information S1, HLA class I category was not a significant independent predictor of mock responses, in addition to Race and Ad5 seropositivity which were found to be associated with mock response. Table S1: Estimates of immune correlate hazard ratios (HR) for HIV-1 infection in vaccine recipients from multivariate Cox regression models of background-unadjusted HIV-specific immune responses. In addition to the ELISpot response variables, all models adjusted for baseline covariates as specified in the Methods section including circumcision status, Ad5 seropositivity, region, race, age, HSV-2 serostatus, recreational drug usage, unprotected receptive anal sex with HIV+ male partner, unprotected insertive anal sex with HIV+ male partner, and number of male partners. Due to missing data in other covariates, 82 (instead of 86) infections were included in these models. Model Foreground indicates antigen-stimulated immune responses without background adjustment. Adjusted indicates antigen-stimulated immune responses subtracted by mock responses. Table S2: Estimates of the effect of ELISpot Mock responses and HLA class I types on risk of HIV-1 infection among vaccine recipients. Table S3: Estimates based on a multivariate linear regression model of the effect of baseline covariates and HLA class I types on week 8 ELISpot mock responses among vaccine recipients. HLA class I type was assessed as an additional independent predictor in the best fitting model that includes race and Ad5 serostatus as presented in Table 3.
(PDF)
