# Supplementary material for: Immune-Correlates Analysis of an HIV-1 Vaccine Efficacy Trial Reveals an Association of Nonspecific Interferon-γ Secretion with Increased HIV-1 Infection Risk: A Cohort-Based Modeling Study
Source: PLoS One. 2014 Nov 4;9(11):e108631. doi: 10.1371/journal.pone.0108631 (PMC4219669; doi:10.1371/journal.pone.0108631)
Supplement: Information S1 — Supporting figures and tables. Figure S1. Week 30 paired ELISpot responses to Gag in the assay comparison study (Panel A & B) and week 8 ELISpot responses to Gag in the Main study before and after calibration (Panel C & D). Panels A & B show the distribution of Gag responses based on the paired samples. One outlier of Gag response was removed before the calibration. Panel C shows the distribution of the 27 bonus samples before and after calibration. Panel D shows the distribution of the Week 8 samples before and after calibration. Figure S2: Distribution of ICS Reponses in Infected (Inf.) and Uninfected (Uninf.) Vaccine and Placebo Recipients. Panel A includes the mock ICS responses. Panel B includes the ICS responses against CMV stimulation. Panel C includes the ICS responses against HIV-1 antigens. Panel D includes the ICS responses against Ad5 antigens. Box plots show the 25th percentile (lower edge of the box), 50th percentile (horizontal line in the box), and 75th percentile (upper edge of the box) for the immune responses, with participants stratified according to HIV-1 infection status and treatment assignment. The tip of the vertical bars indicate the most extreme data points, which are no more than 1.5 times the interquartile range from the box. Figure S3: Correlations between Mock ELISpot Responses and Ad5-specific or CMV-specific ICS Responses among Vaccine Recipients. A fitted simple linear regression line is added with values in the upper corner indicating the Spearman's correlations coefficients (ρ) and p-values (p) from the exact test of ρ being zero. Figure S4: Correlations between the Number of IFN-γ-secreting Cells Measured by Mock ELISpot and the Proportions of IFN-γ-secreting Cells in Different Cell Subsets Measured by Mock ICS among Vaccine Recipients. A fitted simple linear regression line is added with values in the upper corner indicating the Spearman's correlations coefficients (ρ) and p-values (p) from the exact test of ρ being zero. Figure [file pone.0108631.s001.pdf]

Supporting Information

Calibration of immune response measurements between two laboratories

Based on n=494 paired Step study samples collected at week 30 between the Merck and HVTN labs (denoted as lab1 and lab 2 in Figure S1), ELISpot assay results of the same antigen-stimulation at Week 8 from Merck and HVTN were calibrated before they were combined in the described immune correlates analysis. Twenty-seven samples (Main study validation data) at week 8 were also measured by both labs and hence are used to validate the performance of the assay calibration. Supporting Figure 1 illustrates the calibration process for responses against Gag stimulation. Based on Panel C in Figure S1, we observed that the assay calibration process was able to bring the ELISpot measurements from these two labs to a similar level. Similar process was carried out for the calibration of ELISpot mock responses and responses to Pol and Nef. Details of the calibration method can be found in [1].

Figure S1: Week 30 paired ELISpot responses to Gag in the assay comparison study (Panel A & B) and week 8 ELISpot responses to Gag in the Main study before and after calibration (Panel C & D). Panels A & B show the distribution of Gag responses based on the paired samples. One outlier of Gag response was removed before the calibration.   Panel C shows the distribution of the 27 bonus samples before and after calibration. Panel D shows the distribution of the Week 8 samples before and after calibration.

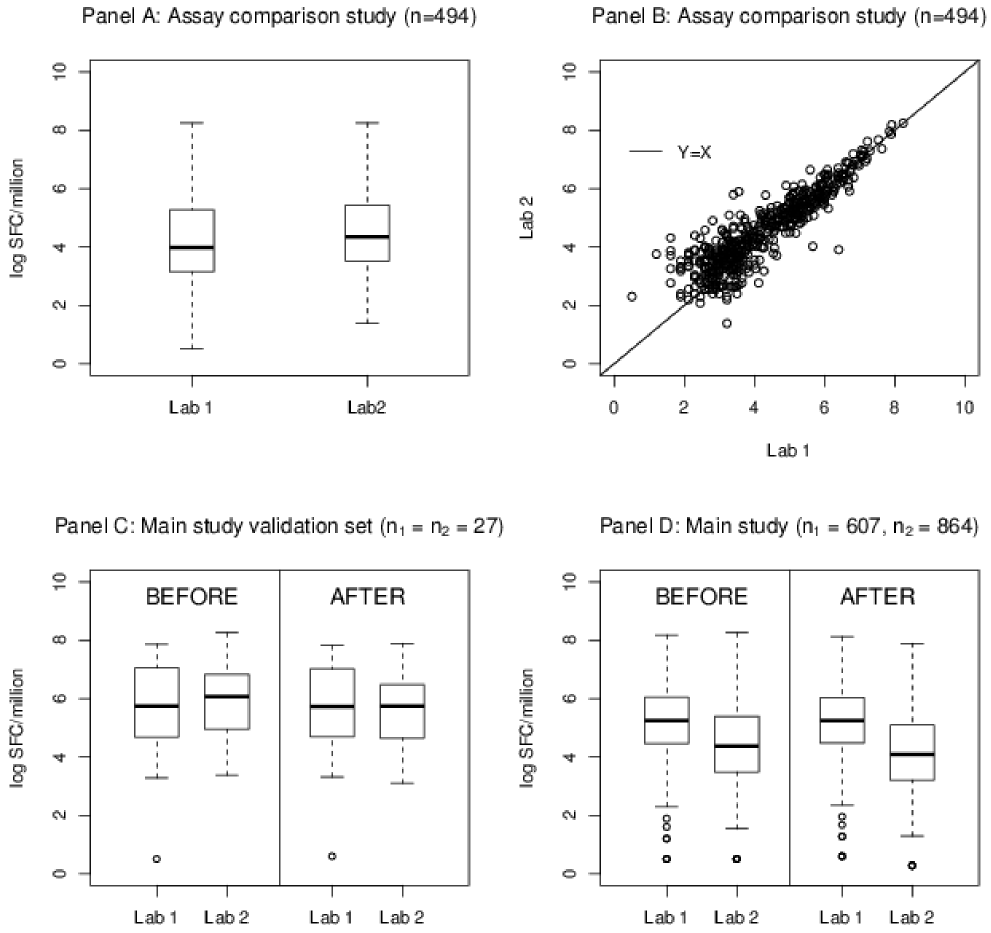

Figure S2: Distribution of ICS Reponses in Infected (Inf.) and Uninfected (Uninf.) Vaccine and Placebo Recipients. Panel A includes the mock ICS responses. Panel B includes the ICS responses against CMV stimulation. Panel C includes the ICS responses against HIV-1 antigens. Panel D includes the ICS responses against Ad5 antigens. Box plots show the 25th percentile (lower edge of the box), 50th percentile (horizontal line in the box), and 75th percentile (upper edge of the box) for the immune responses, with participants stratified according to HIV-1 infection status and treatment assignment. The tip of the vertical bars indicate the most extreme data points, which are no more than 1.5 times the interquartile range from the box.

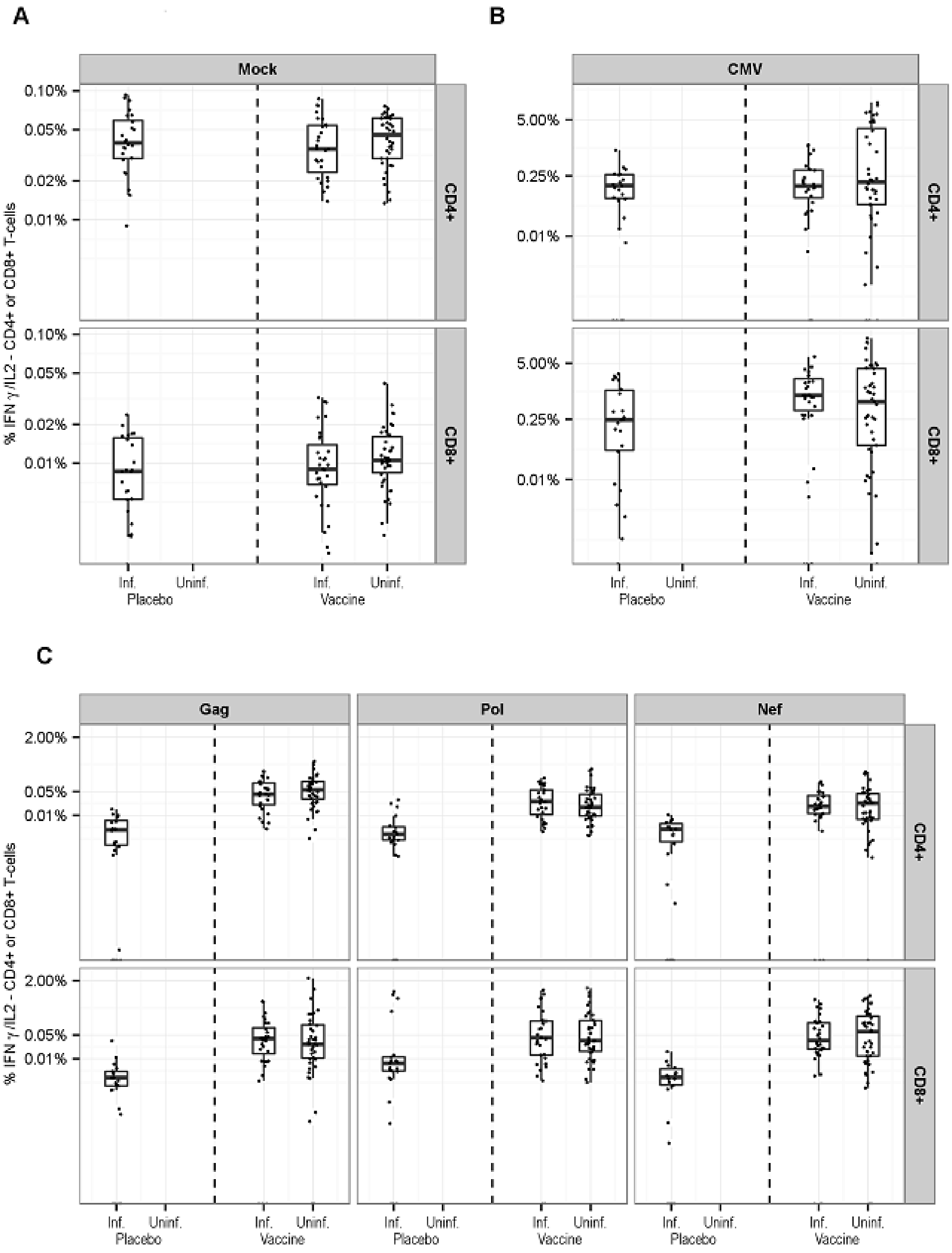

Figure S3: Correlations between Mock ELISpot Responses and Ad5-specific or CMV-specific ICS Responses among Vaccine Recipients. A fitted simple linear regression line is added with values in the upper corner indicating the Spearman's correlations coefficients (ρ) and p-values (p) from the exact test of ρ being zero.

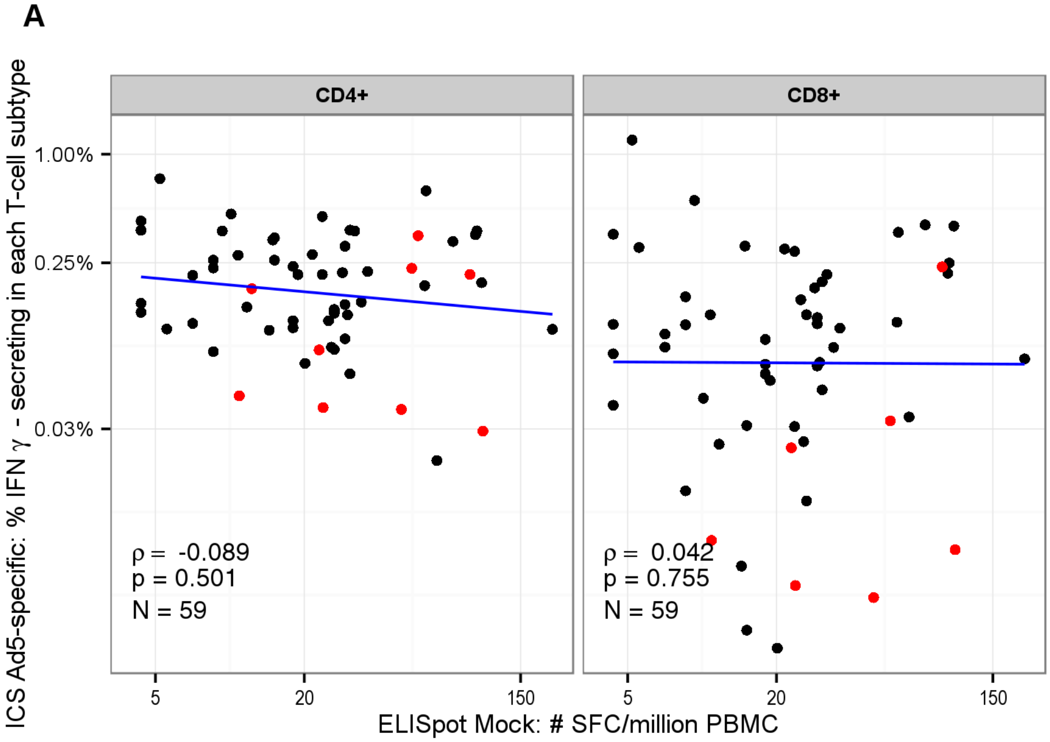

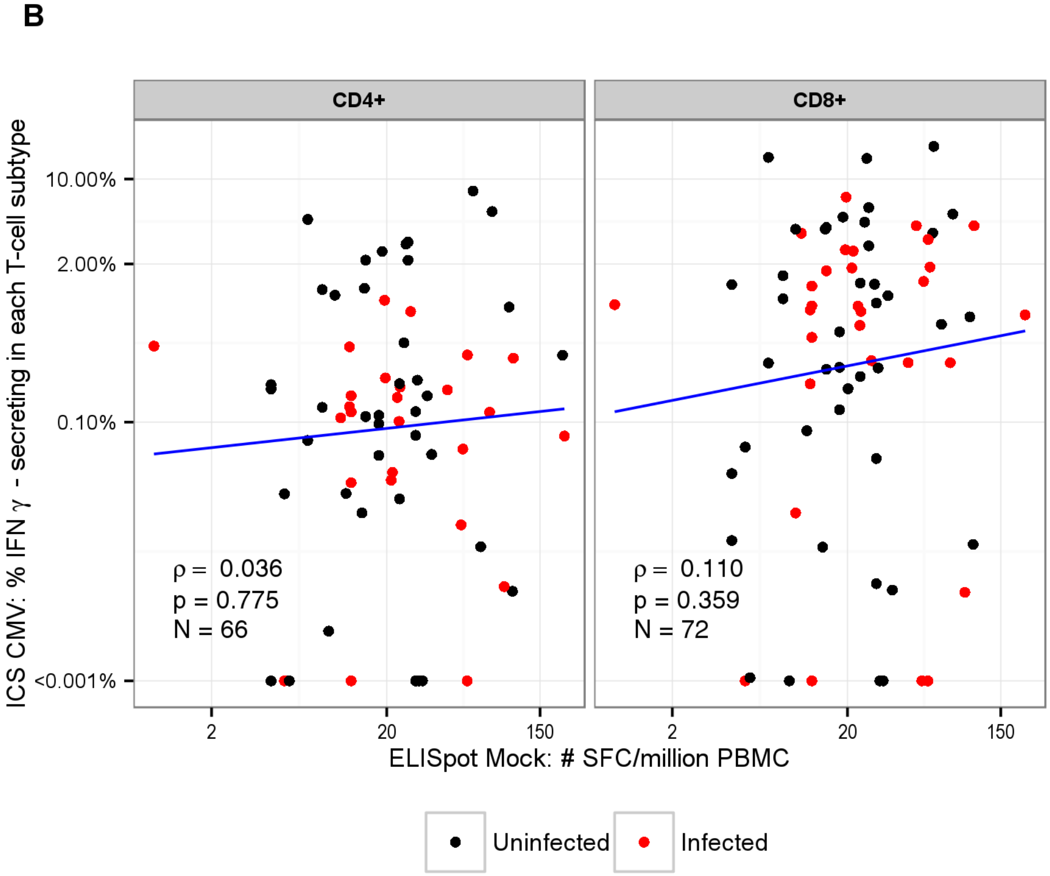

Figure S4: Correlations between the Number of IFNγ-secreting Cells Measured by Mock ELISpot and the Proportions of IFNγ-secreting Cells in Different Cell Subsets Measured by Mock ICS among Vaccine Recipients. A fitted simple linear regression line is added with values in the upper corner indicating the Spearman's correlations coefficients (ρ) and p-values (p) from the exact test of ρ being zero.

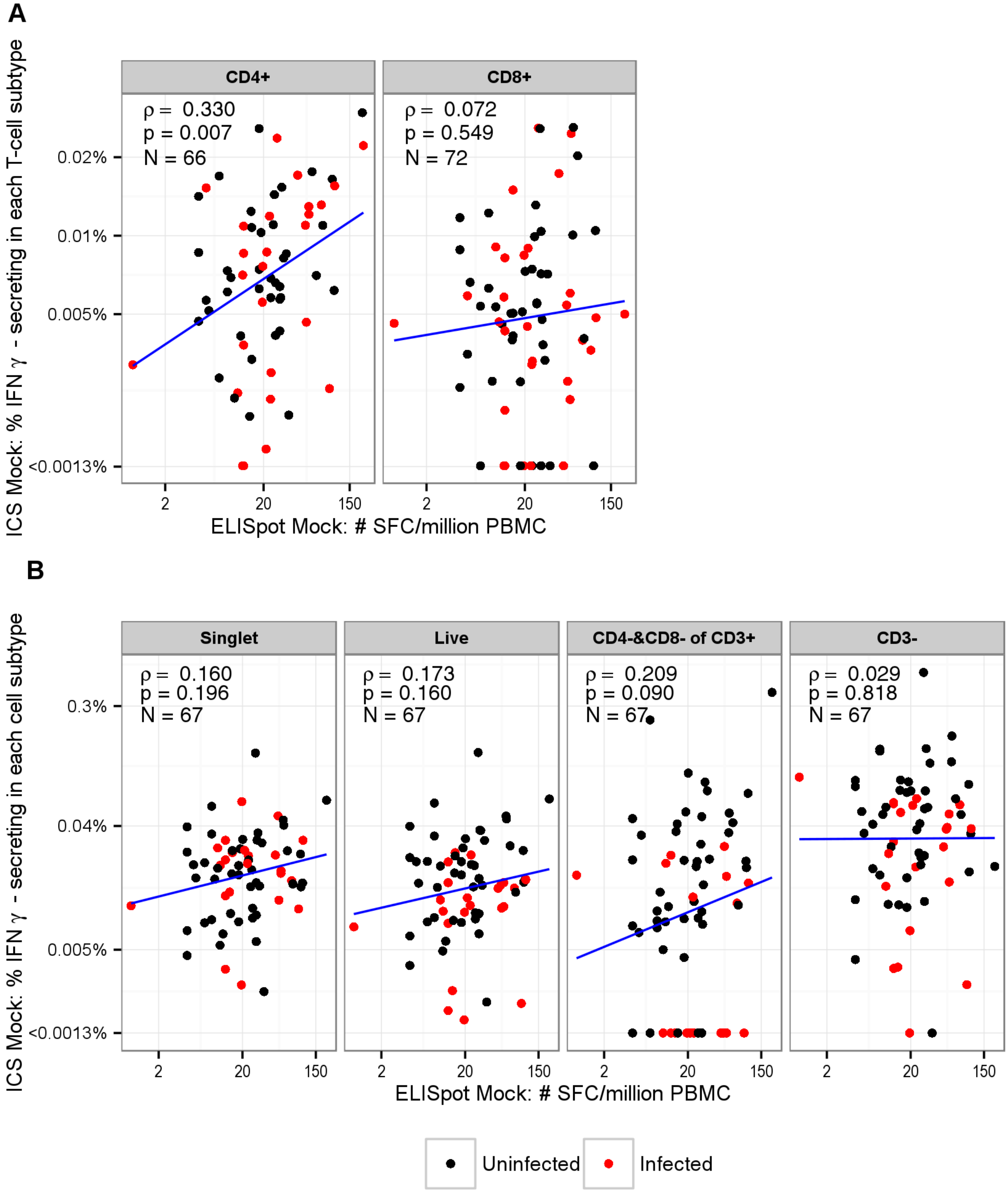

Figure S5: Estimated instantaneous hazard ratios over time per 1-log$_e$ increase of immune responses from each baseline-covariate-adjusted model of a single immune variable as presented in Table 1.

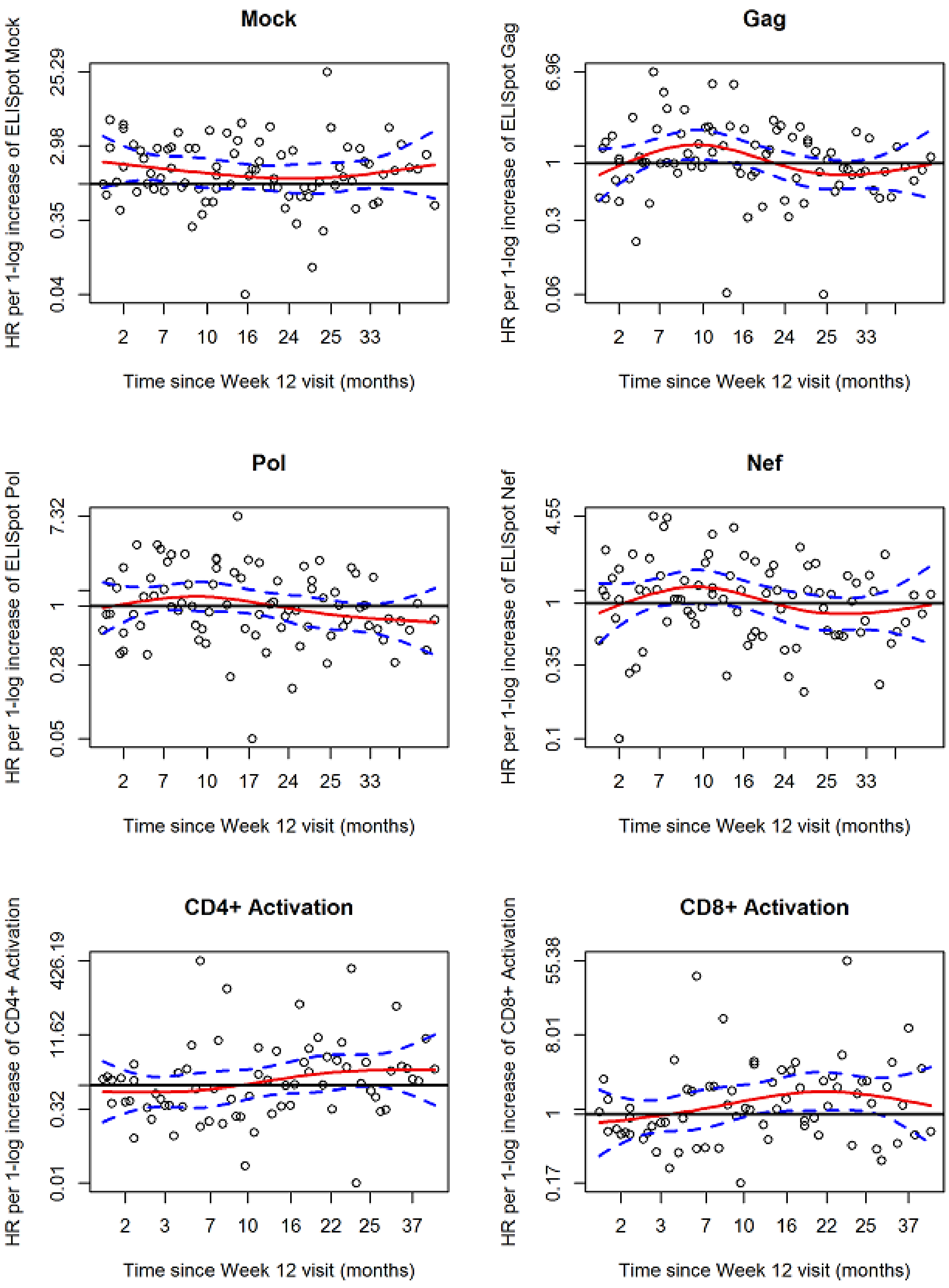

Figure S6: Distribution of Week 8 mock ELISpot responses by HLA class I categories among vaccine recipients. Red dots indicate infected cases and blue dots indicate uninfected non-cases. Mock responses do not seem to differ among different HLA class I categories. As shown in Supporting Table 3, HLA class I category was not a significant independent predictor of mock responses, in addition to Race and Ad5 seropositivity which were found to be associated with mock response.

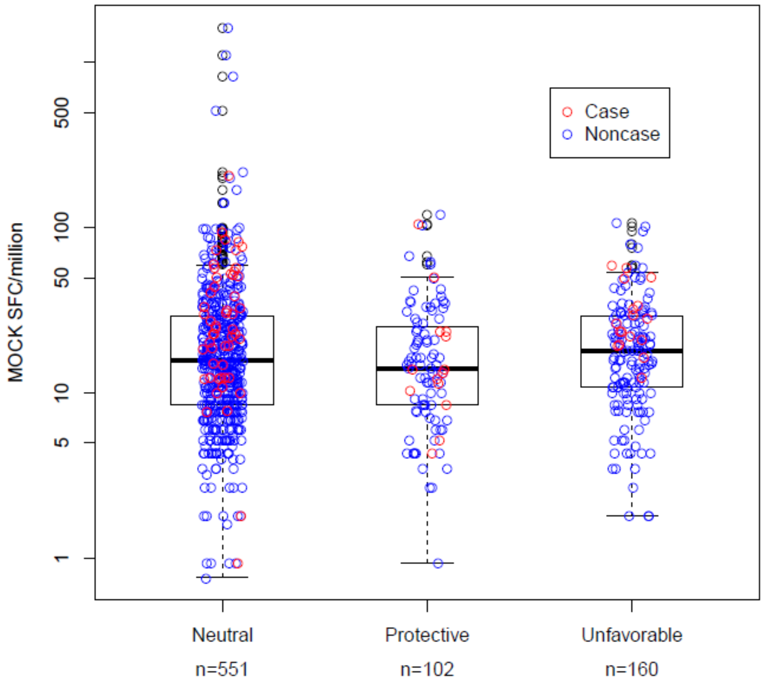

Table S1: Estimates of immune correlate hazard ratios (HR) for HIV-1 infection in vaccine recipients from multivariate Cox regression models of background-unadjusted HIV-specific immune responses. In addition to the ELISpot response variables, all models adjusted for baseline covariates as specified in the Methods section including circumcision status, Ad5 seropositivity, region, race, age, HSV-2 serostatus, recreational drug usage, unprotected receptive anal sex with HIV+ male partner, unprotected insertive anal sex with HIV+ male partner, and number of male partners. Due to missing data in other covariates, 82 (instead of 86) infections were included in these models. Model Foreground indicates antigen-stimulated immune responses without background adjustment. Adjusted indicates antigen-stimulated immune responses subtracted by mock responses.

Immune Variable(s) in Each Model	Immune Variable	HR per 1- $\log_e$ increase (95% CI)	p-value
**Gag-stimulated responses**			
Background-Unadjusted Gag	Gag	1.15 (0.91,1.45)	0.23
Background-Unadjusted Gag + Mock	Gag	0.98 (0.75,1.29)	0.90
	Mock	1.63 (1.26,2.11)	<0.001
**Pol-stimulated responses**			
Background-Unadjusted Pol	Pol	1.09 (0.86,1.38)	0.48
Background-Unadjusted Pol + Mock	Pol	0.94 (0.73,1.21)	0.62
	Mock	1.66 (1.29,2.14)	<0.001
**Nef-stimulated responses**			
Background-Unadjusted Nef	Nef	1.17 (0.94,1.46)	0.16
Background-Unadjusted Nef + Mock	Nef	1.02 (0.79,1.30)	0.90
	Mock	1.61 (1.27,2.04)	<0.001

Table S2: Estimates of the effect of ELISpot Mock responses and HLA class I types on risk of HIV-1 infection among vaccine recipients.

Covariate	HR (95% CI)	p-value
Mock (per one-$\log_e$ increase)	1.63 (1.34, 1.99)	<0.001
HLA (protective vs. neutral)	1.60 (0.89, 2.88)	0.12
HLA (unfavorable vs. neutral)	1.19 (0.68, 2.05)	0.53

Table S3: Estimates based on a multivariate linear regression model of the effect of baseline covariates and HLA class I types on week 8 ELISpot mock responses among vaccine recipients. HLA class I type was assessed as an additional independent predictor in the best fitting model that includes race and Ad5 serostatus as presented in Table 3.

Covariate	Coefficient (95% CI)	p-value	Overall Wald-test p-value
Race (white vs. other)	0.15 (0.006, 0.29)	0.04	
Ad5 serostatus(positive vs. negative)	0.20 (0.06, 0.34)	0.01	
HLA (protective vs. neutral)	-0.13 (-0.33, 0.07)	0.20	0.38
HLA (unfavorable vs. neutral)	0.02 (-0.14, 0.19)	0.77	

References:

1. Huang Y, Huang Y, Moodie Z, Li S, Self S (2012) Comparing and combining data across multiple sources via integration of paired-sample data to correct for measurement error. Stat Med 31: 3748-3759.
